# Indications and Outcomes of Colon Resection in a Conflict Zone: A Single-Center Experience From a Military Hospital in Yemen

**DOI:** 10.7759/cureus.97456

**Published:** 2025-11-21

**Authors:** Ali Albrashi, Yasser A Obadiel, Hussam A Aldomini, Haitham M Jowah

**Affiliations:** 1 Department of Surgery, General Military Hospital, Sana'a, YEM; 2 Department of Surgery, Faculty of Medicine and Health Science, Sana'a University, Sana'a, YEM

**Keywords:** austere environment, colectomy, colon resection, penetrating trauma, surgical outcomes, war surgery, yemen

## Abstract

Background: Although colon resection is a common procedure, data on its indications and outcomes in conflict-affected regions are limited. These environments present unique surgical challenges, driven by the dual burden of acute trauma and advanced-stage chronic diseases. This study aimed to analyze the experience of colon resection to identify key risk factors and inform surgical practices in austere settings.

Methods: A single-center prospective observational study was conducted on 89 patients who underwent colon resection at a military hospital in Sana'a, Yemen, between September 2022 and April 2024. Data on patient demographics, indications, surgical procedures, and postoperative outcomes were collected and analyzed. The primary endpoint was the incidence of adverse outcomes, defined as a composite of significant morbidity and in-hospital mortality.

Results: A total of 89 patients were included (mean age 36.9 years; n=81, 91.0% male). The predominant indication was penetrating trauma (n=70, 78.7%). The overall adverse outcome rate was 19.1% (n=17), comprising a significant morbidity rate of 7.9% (n=7) and an in-hospital mortality rate of 11.2% (n=10). Significant morbidity included events such as anastomotic leak requiring re-operation. Patients presenting with shock on admission had a higher adverse outcome rate than stable patients (34.6% vs. 12.7%); this represented a strong clinical trend that approached statistical significance (OR 3.47, 95%CI 0.95-12.69; P=0.066).

Conclusion: In this conflict-zone hospital, the patient's initial physiological state, particularly the presence of shock, was the most critical determinant of the postoperative outcomes. Despite the austere conditions, the observed anastomotic leak rate suggests that primary anastomosis is a viable option for carefully selected, hemodynamically stable patients. These findings underscore the need for context-specific guidelines that prioritize physiological risk stratification to guide surgical decision-making.

## Introduction

Colon resection is a fundamental surgical procedure, but the evidence base is overwhelmingly dominated by studies from stable, high-income settings [1,2]. A review of the literature reveals a profound scarcity of prospective surgical outcome data from conflict zones. Although established guidelines from high-income settings exist, their applicability in austere environments is uncertain. These guidelines often assume the availability of resources such as 24-hour access to computed tomography (CT), postoperative intensive care unit (ICU) beds, and a reliable supply chain for stoma care products, which are frequently unavailable in conflict settings [3,4]. This gap in evidence undermines the development of context-specific surgical protocols.

Yemen's protracted conflict has devastated its healthcare system, creating the dual burden of surgical disease-the convergence of acute, high-energy trauma, and delayed presentations of malignancy. Hospital-based studies from Yemen confirmed that penetrating injuries from gunshots and explosions are a primary driver of surgical emergencies [5]. A recent prospective study from Sana'a highlighted this reality, showing that penetrating injuries accounted for over 75% of surgically managed colonic trauma cases [6]. Concurrently, disruptions in healthcare have led to cancer care crises. Data from Aden show that 70% of colorectal cancer patients are diagnosed at an advanced stage (Stage III/IV), leading to high rates of emergency presentation for obstruction or perforation [7]. This convergence of acute trauma and complex oncologic disease strains an already fragile system; however, the factors that truly drive outcomes in this setting remain poorly characterized.

Therefore, this study aimed to analyze the indications, surgical management, and postoperative outcomes of patients who underwent colon resection at a single military hospital in Sana'a, Yemen. We sought to identify the primary drivers of adverse outcomes in this high-risk cohort, hypothesizing that initial physiological compromise, specifically hemodynamic instability, would be a more critical determinant of morbidity and mortality than the underlying pathology. By elucidating the realities of colectomy in the conflict zone, our findings may inform context-appropriate surgical practice and improve patient care in similar settings.

This article was previously posted as a preprint on the Research Square platform on May 15, 2024.

## Materials and methods

Study design and setting

This study was a single-center prospective observational analysis of patients undergoing colon resection surgery. All patients were recruited from the surgical department of the General Military Hospital in Sana'a, Yemen. The study period was September 2022 to April 2024. Ethical approval for the study was granted by the Institutional Review Board of the Yemeni Board for Medical Specialization (approval number: IRB/YBMS/2022/031), and administrative approval was obtained from the Military General Hospital. This study was conducted in accordance with the principles of the Declaration of Helsinki. Written informed consent was obtained from all participants or their legal guardians.

Patient population

Patients admitted to the surgical ward who underwent any form of colon resection (hemicolectomy or segmental resection) were eligible for inclusion. All eligible patients who presented during the study period were consecutively recruited to minimize selection bias. To ensure comprehensive enrollment, a screening log was maintained by the on-call surgical team 24/7, and all patients meeting the inclusion criteria were captured, including those presenting after hours. Patients were excluded if they had incomplete medical records that precluded accurate data extraction, were unwilling to participate in the follow-up, or had a terminal illness in which the focus of care was primarily palliative. A total of 89 patients met the inclusion criteria and were enrolled in the study.

Data collection and variables

Data were prospectively collected using a structured questionnaire (Appendix A) supplemented by a thorough review of electronic and paper-based medical records. The questionnaire was newly developed for this study by the authors to capture key variables and was pilot-tested on a small cohort of 10 patients to ensure clarity and completeness before full study implementation. The follow-up period for postoperative outcomes was 60 days. The primary composite endpoint (significant morbidity and in-hospital mortality) was assessed during the index hospitalization, for which complete data were available for all 89 enrolled patients. Post-discharge follow-up data at 60 days were available for 92% (73/79) of the surviving patients, with no new significant morbidity events reported within this timeframe.

The primary variables collected included patient demographics, such as age and sex. Clinical data included complaints, physical examination findings, pre-existing comorbidities, and final diagnosis. For all malignant cases, the diagnosis was confirmed by postoperative histopathological analysis. For all trauma cases, the diagnosis was confirmed by direct intraoperative visualization. Surgical data included preoperative investigations (e.g., CT scan and colonoscopy), use of mechanical bowel preparation, type of surgery (elective, urgent, or emergency), specific surgical procedures performed (e.g., right hemicolectomy), and method of bowel management post-resection (primary anastomosis vs. ostomy). Postoperative outcomes included specific postoperative complications, final clinical outcomes (improved, morbid, or death), and total length of hospital stay.

Outcome measures and definitions

The primary outcome of interest was the incidence of adverse outcomes, defined as a composite of significant postoperative morbidity and in-hospital mortality. Significant morbidity was defined as any complication requiring surgical, endoscopic, or radiological intervention under general anesthesia or resulting in lasting disability. This definition corresponds to a Clavien-Dindo classification of Grade IIIb or higher (Table 1). A detailed mapping of specific complications to their corresponding grades is provided in Appendix B. An "improved" outcome was defined as survival to hospital discharge without significant morbidity. Shock on admission was defined as a systolic blood pressure <90 mmHg or the requirement for vasopressor support.

**Table 1:** Definition of significant morbidity events and mapping to Clavien-Dindo classification Significant morbidity for the primary composite endpoint was defined as any postoperative complication corresponding to a Clavien-Dindo grade of IIIb or higher, which includes complications requiring surgical, endoscopic, or radiological intervention under general anesthesia. Grade I complications require no pharmacological treatment, and Grade II complications require pharmacological treatment (e.g., anticoagulants for deep vein thrombosis).

Complication Event	Definition Used	Corresponding Clavien-Dindo Grade	Classified as "Significant Morbidity"?
Anastomotic Leak	Postoperative leak requiring surgical re-operation and diverting ileostomy.	Grade IIIb	Yes
Necrotizing Fasciitis	Aggressive soft tissue infection requiring surgical debridement under general anesthesia.	Grade IIIb	Yes
Osteomyelitis	Bone infection confirmed on imaging or intraoperatively, requiring surgical debridement.	Grade IIIb	Yes
Surgical Site Infection	Deep or organ/space infection requiring operative drainage under general anesthesia.	Grade IIIb	Yes
Surgical Site Infection	Superficial infection requiring bedside opening of the wound (no anesthesia).	Grade I	No
Deep Vein Thrombosis	Confirmed on ultrasound, requiring therapeutic anticoagulation.	Grade II	No

Surgical decision-making

The decision to perform a primary anastomosis versus an ostomy was at the discretion of the attending surgeon and was guided by the patient's intraoperative findings and physiological status. In general, primary anastomosis is considered for hemodynamically stable patients who do not require massive transfusion and have minimal fecal contamination. Patients presenting with shock, gross fecal contamination, or delayed presentation (>12 hours) were typically managed with resection and ostomy.

Statistical analysis

All data were analyzed using IBM SPSS Statistics for Windows, version 24.0 (IBM Corp., Armonk, New York, United States). Descriptive statistics were used to summarize the cohort characteristics; continuous variables (e.g., age and length of stay) were presented as mean ± standard deviation (SD), while categorical variables were presented as frequencies and percentages. Normality of distribution for continuous variables was evaluated using the Shapiro-Wilk test. For comparative analysis, Fisher's Exact Test was used to assess the associations between categorical variables, given the small sample size. This test was used to compare the rates of adverse outcomes between different patient groups (e.g., elective vs. non-elective surgery; trauma vs. cancer diagnosis). Differences were considered statistically significant at P < 0.05.

## Results

Patient demographics and diagnostic profile

A total of 89 patients who underwent colon resection were included in the final analysis. The cohort was overwhelmingly male (n=81, 91.0%) with a mean age of 36.9 ± 12.1 years. The predominant diagnosis was penetrating abdominal trauma, which accounted for 70 cases (78.7%). Malignancy was the second most common indication, diagnosed in 14 patients (15.7%). Detailed demographic and diagnostic characteristics are summarized (Table 2).

**Table 2:** Patient demographics and clinical characteristics (N=89)

Characteristic	Value
Age (years), mean±SD	36.9±12.1
Sex, n (%)	
Male	81 (91.0%)
Female	8 (9.0%)
Primary Diagnosis, n (%)	
Penetrating Trauma	70 (78.7%)
Cancer	14 (15.7%)
Other Benign Conditions	5 (5.6%)

Surgical procedures and management

Reflecting the acute nature of the presenting pathologies, the vast majority of surgeries were performed on a non-elective basis (n=80, 89.9%). The most common initial surgical strategy was primary anastomosis, which was performed in 55 patients (61.8%). Primary ostomies were performed in 33 patients (37.1%). Right hemicolectomy was the most frequent type of resection (n=33, 37.1%). A summary of surgical management is provided in Table 3.

**Table 3:** Surgical procedures and initial management (N=89)

Procedure / Management	Frequency (Percentage)
Operative Procedure	
Right Hemicolectomy	33 (37.1%)
Left Hemicolectomy	10 (11.2%)
Other Resections	46 (51.7%)
Initial Post-Resection Management	
Primary Anastomosis	55 (61.8%)
Primary Ostomy	33 (37.1%)
Other	1 (1.1%)

Postoperative outcomes and complications

The overall adverse outcome rate was 19.1% (n=17), and the overall mortality rate was 11.2% (n=10). Of the 55 patients who underwent primary anastomosis, four (7.3%) developed a postoperative leak. All four leak cases were managed successfully with surgery, and a diverting ileostomy was performed. The most frequent postoperative complication was necrotizing fasciitis, which was observed in five patients (5.6%). A detailed breakdown of the outcomes and complications is presented (Table 4).

**Table 4:** Postoperative complications and final outcomes (N=89)

Outcome / Complication	Frequency (Percentage)
Final Outcome	
Improved	72 (80.9%)
Adverse (Morbid or Death)	17 (19.1%)
Mortality	10 (11.2%)
Overall Complication Rate	24 (27.0%)
Specific Complications	
Necrotizing Fasciitis	5 (5.6%)
Anastomotic Leak	4 (4.5%)
Surgical Site Infection	4 (4.5%)
Deep Vein Thrombosis	2 (2.2%)
Other Complications	9 (10.1%)

Close examination of the most severe complications revealed important patterns. All four cases of anastomotic leak occurred in hemodynamically stable trauma patients who underwent primary anastomosis. Leaks were diagnosed postoperatively and managed successfully with surgical re-exploration and diverting ileostomy. The five patients who developed necrotizing fasciitis also had penetrating trauma; one of these cases resulted in death. These findings highlight that even in patients who are hemodynamically stable on admission, the severity of the underlying tissue injury in a conflict zone poses a significant risk of major complications.

Outcomes in the trauma-only cohort

To provide a focused analysis on conflict-related injuries, we examined the outcomes specifically for 70 patients with penetrating trauma. Within this subgroup, the adverse outcome rate was 20.0% (n=14), and the in-hospital mortality rate was 12.9% (n=9). Primary anastomosis was performed in 42 of these trauma patients, with three (7.1%) developing a postoperative anastomotic leak. The most frequent significant complication in the trauma cohort was necrotizing fasciitis (n=4, 5.7%).

Comparative analysis of risk factors for adverse outcomes

Comparative analyses were performed to identify risk factors associated with adverse outcomes. Hemodynamic status on admission was the most powerful predictor. Patients presenting with shock had an adverse outcome rate of 34.6% (9/26), compared to 12.7% (8/63) in hemodynamically stable patients. This represented a more than three-fold increase in the odds of an adverse outcome (OR 3.47; 95%CI 0.95-12.69), a strong clinical trend that approached statistical significance (P=0.066). In contrast, no statistically significant associations with adverse outcomes were found for other variables. The adverse outcome rate for trauma patients was 20.0% compared to 7.1% for cancer patients (OR 3.25; 95%CI 0.40-26.35; P=0.428). Similarly, there was no significant difference between non-elective and elective surgeries (20.0% vs. 11.1%; OR 1.97; 95%CI 0.24-16.35; P=1.000). The results are summarized in Table 5.

**Table 5:** Comparative analysis of factors associated with adverse outcomes (N=89) ORs and p-values were calculated using Fisher's Exact Test. The reference group for each OR calculation is also indicated.

Factor Group	Adverse Outcome, n (%)	Improved Outcome, n (%)	Total	Odds Ratio (95% CI)	p-value
Hemodynamic Status					0.066
Not in Shock (Reference)	8 (12.7%)	55 (87.3%)	63	1.00	
Shock	9 (34.6%)	17 (65.4%)	26	3.47 (0.95 – 12.69)	
Diagnosis Type					0.428
Cancer (Reference)	1 (7.1%)	13 (92.9%)	14	1.00	
Trauma	14 (20.0%)	56 (80.0%)	70	3.25 (0.40 – 26.35)	
Surgery Urgency					1.000
Elective (Reference)	1 (11.1%)	8 (88.9%)	9	1.00	
Non-Elective	16 (20.0%)	64 (80.0%)	80	1.97 (0.24 – 16.35)	

## Discussion

This study provides a rare and valuable analysis of colon resection in a conflict-zone military hospital, offering critical insights into surgical realities in an austere environment. These findings confirm that penetrating trauma is the overwhelming driver of this procedure; however, more importantly, they reveal crucial clinical insights: a patient's initial physiological state, specifically the presence of shock, is the most powerful predictor of adverse outcomes.

The most clinically significant finding from our analysis was the profound impact of hemodynamic status on patient outcomes. Patients presenting with shock were more than three times more likely to suffer a major complication or death (OR, 3.47), a finding that approached statistical significance (P=0.066). This aligns with the bedrock principles of trauma surgery, where physiological derangement, the "lethal triad" of acidosis, hypothermia, and coagulopathy, is a more potent driver of mortality than the anatomical extent of the injury itself [8]. This evidence strongly supports the use of a stratified surgical approach, as summarized in our institutional algorithm (Appendix B). For hemodynamically unstable patients, damage control surgery, which prioritizes rapid contamination control and physiological resuscitation over definitive, lengthy repair, should be the default strategy to mitigate the high risk of mortality [9].

A central and somewhat unexpected finding of our study was the anastomotic leak rate of 7.3% (n=4) in 55 patients who underwent primary anastomosis. This outcome is highly encouraging. In high-resource settings, the reported leak rates after surgery for penetrating colon trauma range from 5% to 14% [10]. Our anastomotic leak rate of 7.3% is comparable to benchmarks from these settings, such as 6.6% reported in the landmark American Association for the Surgery of Trauma (AAST) multicenter study [11]. This finding is particularly favorable given the high-risk profile of our cohort, in which 29% of the patients presented with shock. This rate of hemodynamic instability is substantially higher than that typically reported in civilian trauma registries, suggesting that achieving a low leak rate in a sicker patient population represents a significant success for selective anastomosis in an austere environment. This provides crucial, context-specific evidence for surgeons operating in similar environments who must weigh the risks of a leak against the known morbidity and resource burden of creating a stoma.

Our analysis did not find a statistically significant difference in adverse outcomes between patients with trauma (n=14, 20.0%) and cancer (n=1, 7.1%). This does not imply that trauma is without risk; rather, it likely reflects the high-risk nature of our non-trauma cohort, where cancer patients often present late with obstructive emergencies, making them physiologically compromised, like many trauma victims [12]. The overall complication rate remains high, and the incidence of necrotizing fasciitis (n=5, 5.6%) is a disturbing outlier that underscores the challenges of managing heavily contaminated wounds in this environment [13].

Limitations

This study had several important limitations. First, our primary finding-that hemodynamic shock is a key predictor of adverse outcomes-is based on a strong clinical trend (P=0.066) that did not reach statistical significance and therefore requires validation in larger, multicenter studies. Second, the single-center design limits the generalizability to other conflict settings with different injury patterns or resources. Third, there was a significant potential for selection bias in the observed anastomotic leak rate. The favorable 7.3% rate likely reflects prudent surgeon selection for primary anastomosis in hemodynamically stable patients, and this rate cannot be generalized to all trauma patients, particularly those in shock. Fourth, our analysis did not include key unmeasured confounding variables, such as injury severity scores (ISS), intraoperative blood transfusion volumes, or operative times. These factors are likely higher in patients presenting with shock and could independently predict adverse outcomes. Finally, the short follow-up period of 60 days precludes the assessment of important long-term outcomes, such as stoma reversal rates or chronic pain.

## Conclusions

In the challenging context of a conflict-zone military hospital, this study suggests that a patient's initial physiological state, particularly the presence of shock, is the most critical predictor of postoperative outcomes. Our findings provide valuable context-specific evidence that primary anastomosis can be a safe and viable option in carefully selected hemodynamically stable patients, thereby challenging the traditional dogma of mandatory diversion in austere settings.

Future surgical guidelines for such environments must prioritize protocols for rapid resuscitation and physiological risk stratification to guide crucial decisions regarding primary repair, damage control, surgery, and diversion. We recommend the development of these context-adapted protocols and further validation of these findings through multicenter data collection in similar conflict zones.

## Figures and Tables

**Figure 1:**
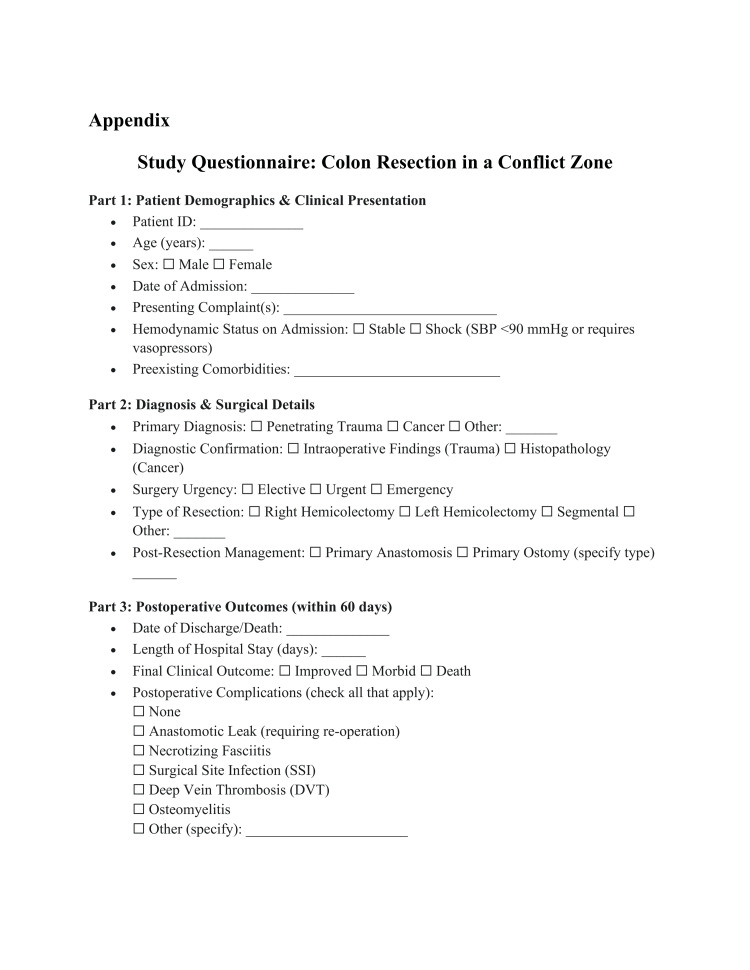
Srudy questionnaire

**Figure 2:**
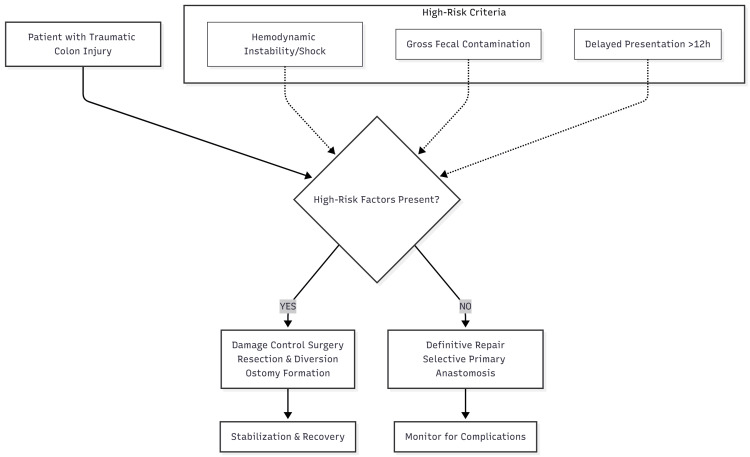
Surgical decision algorithm for management of traumatic colon injuries in an austere setting This algorithm summarizes the institution's stratified approach to managing traumatic colon injuries. The decision between definitive repair (selective primary anastomosis) and damage control surgery (resection and diversion/ostomy) is primarily guided by the patient's initial physiological state and the presence of key high-risk factors, including hemodynamic instability (shock), gross fecal contamination, and delayed presentation (>12 hours). This approach aims to mitigate the high risk of anastomotic complications in physiologically compromised patients while avoiding the morbidity of a stoma in carefully selected, stable patients.

## Appendices

Appendix A 

Appendix B 
